# Clinical and endoscopic characteristics of diffuse esophageal intramural pseudo-diverticulosis

**DOI:** 10.1007/s10388-020-00729-6

**Published:** 2020-03-11

**Authors:** Florian Hentschel, Stefan Lüth

**Affiliations:** 1 Center for Internal Medicine II, Brandenburg Medical School Theodor Fontane, Brandenburg an der Havel, Germany; 2 Zentrum für Innere Medizin II, Hochschulklinikum Brandenburg der MHB, Hochstr. 29, 14770 Brandenburg an der Havel, Germany

**Keywords:** Rare diseases, Esophagus, Pseudodiverticulosis, Endoscopy, Esophageal inflammation, Candidiasis

## Abstract

**Introduction:**

With 250 published cases worldwide, diffuse esophageal intramural pseudo-diverticulosis (DEIPD) is a poorly understood disease. The aim of this study was to determine the prevalence of DEIPD in our own population, identify risk factors and clinical symptoms, and characterize its typical endoscopic signs.

**Methods:**

Retrospective search in our center’s endoscopic and clinical database. Reviewing of all cases by re-examining stored endoscopic photographs. Reviewing of all cases regarding age, sex, risk factors, comorbidities, histology, and clinical symptoms.

**Results:**

In a population of 150.000 we found 21 cases of DEIPD. Mean age was 56 ± 10 years. 86% were males, 76% had alcohol abuse, 57% had nicotine abuse, 38% had arteriosclerosis, 33% had COPD, 29% had malignancies, 24% had liver cirrhosis, 19% had impaired kidney function, and 15% had diabetes. Dysphagia was present in 62% and food bolus impaction (single or repeated) in 48%. Endoscopically, 95% of patients had multiple (> 4), small (0.25–2.5 mm) pseudodiverticle openings in the esophageal wall. In 62%, openings were aligned longitudinally. 86% showed edematous swelling of mucosa (“frosted glass look”), 76% showed a fine-grained pattern of small (10–100 µm) red dots (“faux uni pattern”), and 76% had a rigid, narrow lumen with multiple rings (“trachealization”).

**Conclusion:**

With a prevalence of approximately 5 to 50/100.000, DEIPD may be more frequent than previously estimated. It preferably affects middle-aged male alcoholics. Key symptoms are chronic dysphagia and food impaction. Typical endoscopic findings are multiple, small, longitudinally aligned pseudodiverticle openings, frosted glass look, faux uni pattern, and trachealization of the esophagus.

**Electronic supplementary material:**

The online version of this article (10.1007/s10388-020-00729-6) contains supplementary material, which is available to authorized users.

## Introduction

Diffuse esophageal intramural pseudo-diverticulosis (DEIPD) is a rare disease characterized by chronic inflammation and scarring of the esophagus. Histologically, it is characterized by nonspecific mucosal or submucosal inflammation, with a mixed infiltrate and no preference for lymphocytes or eosinophils. Transmural autopsy specimens show submucosal fibrosis and dilated excretory ducts of submucosal glands, forming the typical, small pseudodiverticula [1, 2].

Clinical signs are unspecific. Most common is dysphagia of varying frequency. Less consistently reported are symptoms like odynophagia, chest pain, weight loss, bolus impaction, and occasional bleeding. In many cases, symptoms persist for several years [3–6].

Therapeutic options are sparse. Endoscopic dilatation of strictures will give temporarily relief from dysphagia, and there are reports of beneficial effects of sucralfate [7, 8]. Antifungal treatment can be helpful, though the significance of candidiasis is unclear [9, 10]. Still, the disease typically takes a chronic course with frequent relapses, the reason for it being unknown.

From the 1960s to the 1990s, DEIPD had been considered an extremely rare disease [11–14]. Incidence in pre-selected patients undergoing esophageal contrast radiography was between 0.15 and 0.26% [15, 16]. More recent endoscopic studies from single tertiary centers found 22 to 23 patients within 10 to 12 years [3, 4].

The pathomechanism leading to pseudodiverticulosis is not known. Alcohol and tobacco abuse are suspected risk factors, but their relevance is unclear. Reported rates of alcoholism in DEIPD patients range from 15.5 to 100% [4, 14]. Other conditions that may be linked to esophageal pseudodiverticulosis include diabetes mellitus, gastro-esophageal reflux (GERD), candida infection, and eosinophilic esophagitis (EoE) [3, 14, 17]. A raised incidence of esophageal malignancies in DEIPD patients is suspected but—due to the small number of known cases—not proven statistically [14, 15, 18, 19].

Until the 1990s, diagnosis of DEIPD was made radiologically, with barium contrast fluoroscopy being method of choice [11, 20]. Since then, radiography has been superseded by flexible video endoscopy [3, 4]. Still, there are no agreed-upon endoscopic criteria for DEIPD, and every author uses his or her own definition [3, 17, 21]. Only very recently, typical endoscopic findings (as well as therapeutic options) in DEIPD were described in a group of 23 patients, one of the largest so far [4].

Against that background, we conducted this study with the following goals:

- Estimating incidence and prevalence of DEIPD in our own pre-selected collective of inpatients and outpatients who underwent esophago-gastro-duodenoscopy (EGD)
- Extrapolate these numbers to the prevalence in the overall population.
- Verify or falsify suspected risk factors for DEIPD
- Describe “typical” DEIPD patients regarding age, sex, and comorbidities
- Assess frequency and severity of typical symptoms
- Systematically describe endoscopic criteria for a definite diagnosis.

## Methods

### PubMed and database search

In February 2020, we conducted a PubMed search for “pseudodiverticulosis AND esophagus”.

We furthermore performed a retrospective search for suspected DEIPD cases in our own center’s endoscopic database (ViewPoint 5.6 SP27, GE Healthcare, Chalfont St Giles, UK). This database contains information about every endoscopy performed in our institution, including indication, sedation, endoscopes used, findings, diagnoses, and stored endoscopic photographs. The Center for Internal Medicine II Brandenburg is a tertiary endoscopic center for a city of 75.000 and the surrounding rural counties, adding to approximately 150.000 inhabitants.

We first searched for EGDs performed between January, 2008 and May 2019. Within these, we then searched for word fragments like “pseudo*”, or “divertic*” or “esophagitis” in the “indication”, “anamnesis” and “diagnosis” data fields. The resulting cases were re-examined by viewing the stored endoscopic photographs. Corresponding anamnestic and clinical data as well as histopathologic reports were extracted from the clinical information system (Medico Release 25.01.10.01, Cerner Health Services GmbH, Idstein, Germany).

### Endoscopy

Endoscopy was performed using standard flexible videogastroscopes (Fujinon EG Series, Fujifilm Holdings K.K., Tokyo, Japan and Olympus GIF series, Olympus K.K., Tokyo, Japan). Nurse-administered sedation (NAPS) was performed using propofol (Propofol 1%, Fresenius Kabi AG, Bad Homburg, Germany) with or without added midazolam (Midazolam 1 mg/1 ml, B. Braun Melsungen AG, Melsungen, Germany) according to current national guidelines [22].

### Histology

In all but two patients, mucosal biopsies were taken from different locations in the upper, middle, and lower esophagus using 2.3 mm calipers (MTW Wolfgang Haag KG, Germany) through flexible gastroscopes (Fujinon EG-600WR or EG-600ZW, Fuji Corp, Japan). Probes were fixated in 4% buffered formaldehyde (R. Langenbrinck GmbH, Emmendingen Germany) and brought to the pathologist’s laboratory. They were then embedded in 10% paraffin wax (Tissue Tek, Sakura Finetek Europe B.V., Netherlands), cut to 4 μm slices (Microtome SM2000R/SM2010R, Leica, Germany), and underwent standard H&E or PAS staining (Hämalaun Mayer and Hämatoxylin Gill III, Dr. K. Hollborn & Söhne GmbH & Co KG, Germany; Erythrosin, Carl Roth GmbH + Co. KG, Germany) in an automated slide stainer and coverslipper (TCA 44-720, MEDITE GmbH, Germany). After routine histopathologic assessment by a specialist in pathology, they were archived.

### Statistics

All numbers were processed using Microsoft Excel 2013 and/or IBM SPSS Statistics 23. Because of the data structure (see discussion), only descriptive statistics was applied.

## Results

### Epidemiology, risk factors, additional diagnoses

The PubMed search for “pseudodiverticulosis AND esophagus” yielded 121 publications, mostly case reports and small series. Adding up all patients from these studies, we calculated a number of approximately 250 published cases worldwide.

**Fig. 1:**
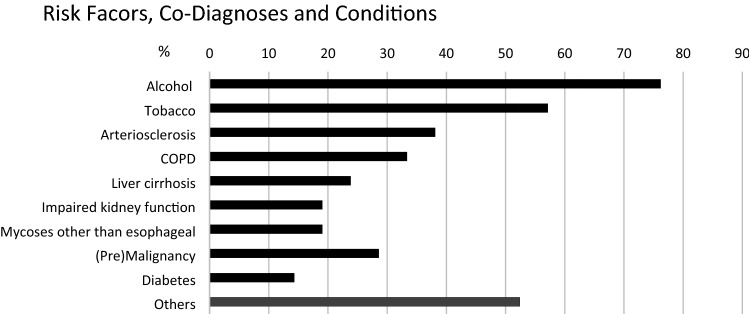
Risk factors, co-diagnoses and conditions in patients with diffuse esophageal intramural pseudodiverticulosis. Most frequent risk factors in patients with DEIPD were alcohol and tobacco abuse with corresponding diagnoses like arteriosclerosis, COPD, and liver cirrhosis. Six out of 21 patients had a malignant tumor or premalignant condition. Four patients had mycosis other than esophageal (mainly dermatitis)

Searching our own endoscopy database for a time span between January 2008 and May 2019, we found 15,096 patients who underwent a total of 24,559 EGD procedures. Within these, 21 patients met the criteria of diffuse chronic esophagitis with pseudodiverticulosis. 18 were males, 3 were females. Mean age was 56 years ± 10 years standard deviation (SD). Mean body mass index (BMI) at first presentation was 25.1 ± 5.3 SD. Of these 21 patients, 16 (76%) had past or ongoing alcohol abuse; 12 (57%) had past or ongoing nicotine abuse (Fig. 1). No data about alcohol consumption was collected in five cases (24%), no data about smoking in nine cases (43%).

Additional diagnoses included eight cases of peripheral or coronary arteriosclerosis (38%), seven cases of COPD (33%), five cases of liver cirrhosis (24%), and four cases of impaired kidney function (19%). Six Patients (29%) had malignant tumors or premalignant conditions, including two cases of well-differentiated neuroendocrine tumors (NET G1) of the stomach and duodenum, one monoclonal gammopathy of undetermined significance (MGUS), one squamous cell carcinoma of the oropharynx, one history of breast cancer, and one basalioma. Diabetes mellitus type I was present in one case (5%), type II in two (10%). Other additional diagnoses included tuberculosis, pancreatitis, atopic dermatitis, rheumatoid arthritis, immobility with pressure ulcer, congestive heart failure, and myocarditis (Fig. 1). Eleven patients (52%) had five or more additional diagnoses not related to the esophagus, 3 patients (14%) had ten or more (min diagnoses 0, max 17, mean 5 ± 4 SD). All patients but one took more than one drug as long-term medication (min drugs 0, max 14, mean 6 ± 4 SD). These included clopidogrel, aspirin, ramipril, metoprolol, spironolactone, hydrochlorothiazide, xipamide, pregabalin, tiotropium bromide, formoterol, corticosteroids, and insulin.

### Symptoms

At initial presentation, 11 patients (52%) reported dysphagia or odynophagia; two (10%) developed dysphagia later on.

Eight patients (38%) underwent emergency endoscopy for food impaction as initial symptom; three of these patients had between two and four repeated impactions later on. (One patient had food impaction 10 years after first complaints of dysphagia). This adds to a total of 18 food impactions in 21 patients over 11 years.

Four patients (19%) reported subjective weight loss in the weeks before first presentation—two of them (10%) because of dysphagia, two because of other reasons. In six cases, weight loss was quantified later during the course of the illness with a median of − 7 kg (min^−1^ kg max − 19 kg, SD = 7).

Two patients (10%) complained about vomiting and/or regurgitation. One (5%) had non-cardiac chest pain and heartburn independent from swallowing (Table 1).

**Table 1:** Clinical symptoms in diffuse esophageal intramural pseudodiverticulosis

Patient	Age	Sex	Dysphagia, odynophagia	Chest pain	Regurgitation, vomiting	Weight loss	Bolus impaction (times)
1	64	m	+	0	0	0	+ (2)
2	55	m	0	0	0	0	0
3	44	m	0	0	0	0	0
4	61	m	0	+	0	0	0
5	72	m	+	0	0	+	+ (1)
6	56	m	+	+	0	0	0
7	56	m	+	0	0	+	+ (3)
8	55	f	+	0	0	0	+ (2)
9	54	m	+	0	0	+	+ (1)
10	31	m	+	0	+	+	0
11	77	m	+	0	0	0	0
12	52	m	0	0	0	0	+ (1)
13	50	m	0	0	+	0	0
14	55	m	+	0	0	+	+ (1)
15	58	m	0	0	0	+	0
16	58	m	+	+	0	0	+ (5)
17	53	m	+	0	0	0	+ (1)
18	84	f	+	0	+	+	0
19	51	m	+	0	0	0	+ (1)
20	63	f	0	0	0	0	0
21	57	m	0	0	0	+	0
Total			13	3	3	8	10^a^
%			62	14	14	38	48^a^

Main symptoms in DEIPD were dysphagia/odynophagia, bolus impaction and weight loss. Less frequent were regurgitations, vomiting, and non-cardiac chest pain. Three patients had no subjective esophagus-related symptoms at all; DEIPD in these patients was an incidental finding during endoscopy for other reasons

^a^Four patients had more than one bolus impaction; each of these patients was counted as one case (+)

### Visits, procedures, and hospital stay

Five patients were outpatients for endoscopy only. 17 patients were admitted to the hospital at least once and stayed at least one night because of esophagitis or pseudodiverticulosis-related symptoms (see below). Eight patients were admitted once, eight patients were admitted between two and five times, one patient was admitted six times. Length of hospital stay was 2 days in 11 cases, 3–6 days in five cases, and 17 days in one case, adding to a total of 57 days in 42 months. For patients that attended more than once, median time between first and last visit (or hospital admittance) for pseudodiverticulosis was 24 months (min 2, max 42, SD = 16).

Additionally, all 21 patients were admitted to the hospital for other reasons than DEIPD at least once. Between August 2008 and August 2019, median time of hospitalization for other diagnoses was 11 days per patient (min 0 max 131), adding to a total of 576 days for 21 patients within 11 years.

Five patients had only one EGD procedure, and one had 12. Median of EGD procedures per patient was 4 (± 3 SD), adding to a total of 83 EGDs in 21 patients within 11 years. Time span between first and last pseudodiverticulosis-related EGD was 12 months median (min = 0, max = 114, SD = 36).

### Endoscopic features

**Fig. 2:**
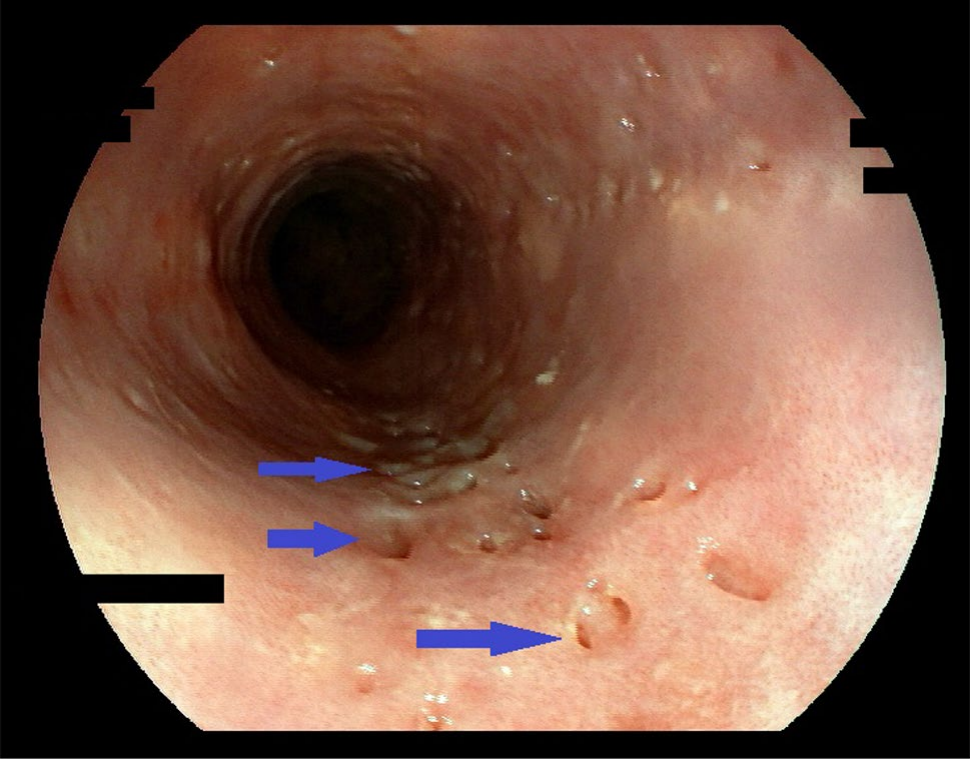
Diverticle openings, faux-uni pattern—endoscopic view. Endoscopic view of DEIPD. Multiple pseudo-diverticle openings throughout the length of the esophagus (arrows). Their longitudinal alignment corresponds with the physiologic distribution of submucosal glands. Patient 16 from Tables 1 and 2; Fujinon EG-600WR, VP-4450HD

Key feature of EIPD are multiple (more than 4) diverticle openings in the esophageal wall. They are small, approximately 0.25–2.5 mm in size, and sometimes open and close synchronous to peristalsis or breathing. They can, therefore, easily be overlooked when the esophagus is viewed in a hurry and/or through an older low-resolution endoscope (Figs. 6a, b). Diverticles can be scattered throughout the organ or, more often, be aligned parallel to the longitudinal axis (Fig. 2 and Online Resources 1 and 2). If esophageal inflammation is present, a cloudy white liquid may be oozing out of these openings (Online Resource 3). In the case of severe inflammation, multiple openings can also merge into longitudinal streaks that resemble the “furrows” characteristic for EoE.

Twenty out of 21 patients (95%) had 5 or more diverticle openings in the esophagus. In 14 patients (67%), these openings were aligned longitudinally.

**Fig. 3:**
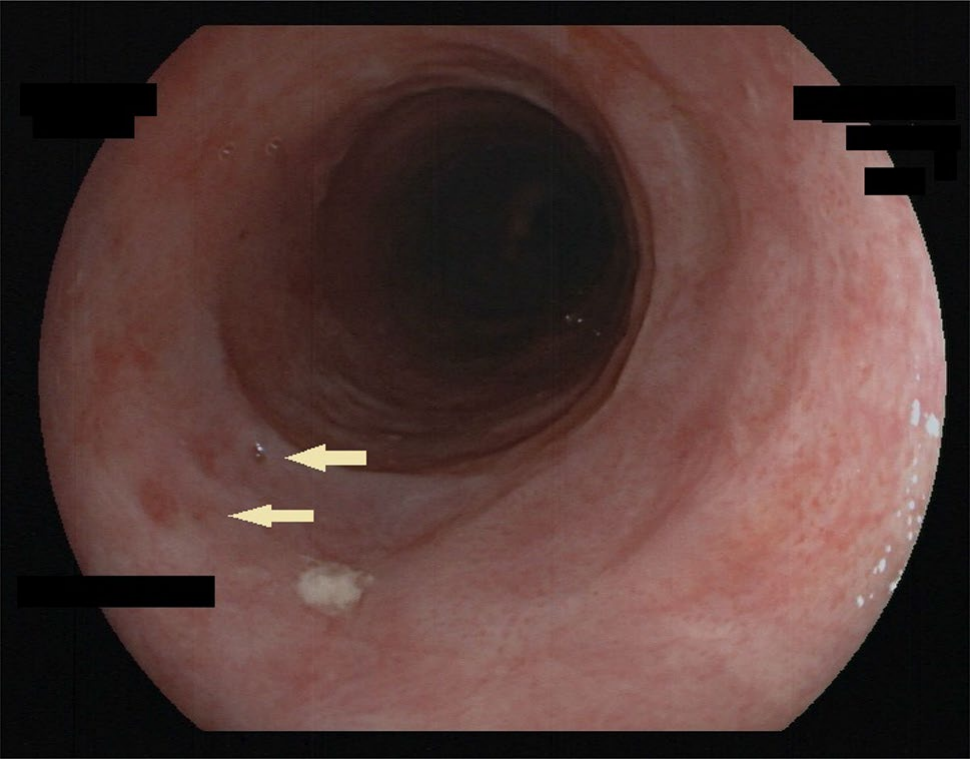
Faux uni pattern—endoscopic view. Endoscopic view of DEIPD, faux-uni pattern: Multiple very small red dots on a light grey esophageal mucosa in the foreground. Discrete ring formation in the middle ground and background. Two bigger red spots in the foreground (arrows) might be early stages of pseudodiverticle formation. Patient 7 from Tables 1 and 2; Fujinon EG-600ZW, VP-4450HD

Another characteristic finding in DEIPD is a fine-grained pinkish reddening of the mucosa between diverticles. A closer look through a high-definition endoscope reveals that this pink tint consists of multiple red dots approximately 10–100 µm in size. In absence of a better name we call this a “faux-uni” pattern, similar to the term used for ultra-fine patterns in fabric (Figs. 3 and 6a, Online Resource 4).

**Fig. 4:**
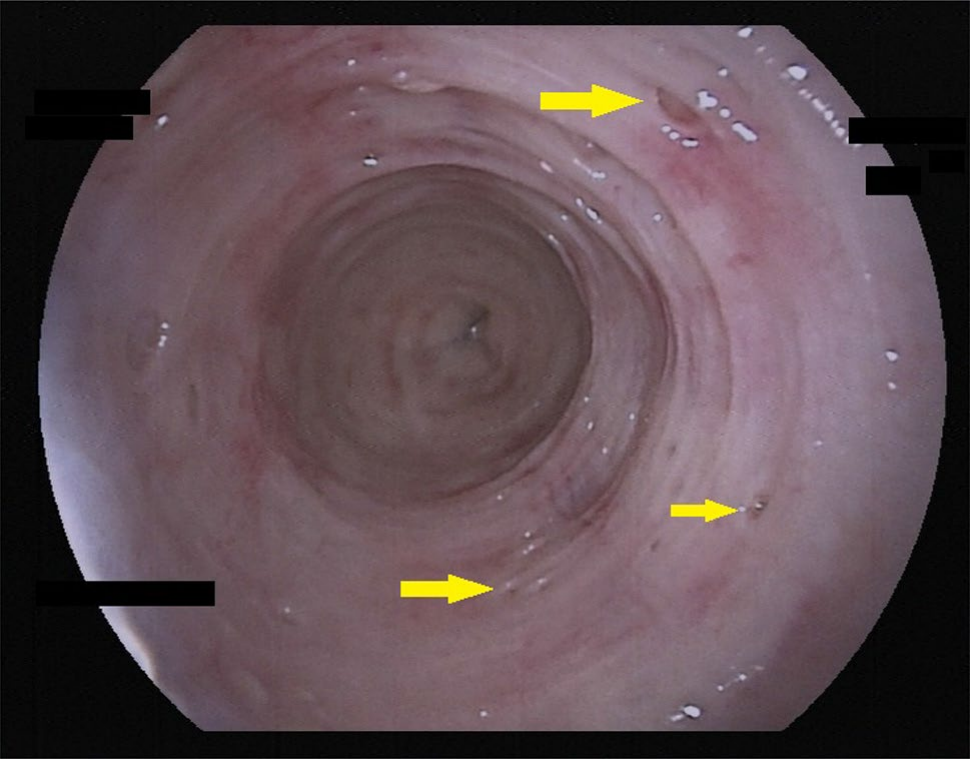
Frosted glass look. Endoscopic view of DEIPD, “frosted glass look” and “trachealization”: We suspect edematous mucosal swelling to be the reason for the characteristic dull-white appearance that resembles frosted glass. Multiple diverticle openings (arrows). With multiple rings and no peristalsis, the esophagus resembles the bronchoscopic view of a trachea. Patient 1 from Tables 1 and 2. Fujinon EG-600WR, VP-4450HD

During active inflammation, faux-uni pattern may vanish and give way to a dull white edematous swelling of the mucosa that resembles frosted glass. This “frosted glass look” can be concentrated around diverticle openings, or be spread over the whole esophageal wall (Fig. 4, Online Resource 5).

Sixteen of 21 patients (76%) showed mucosal faux-uni pattern at least in one EGD, and 18 (86%) showed frosted glass look. Patients with “frosted glass” mucosa also had macroscopic signs of candidosis.

**Fig. 5:**
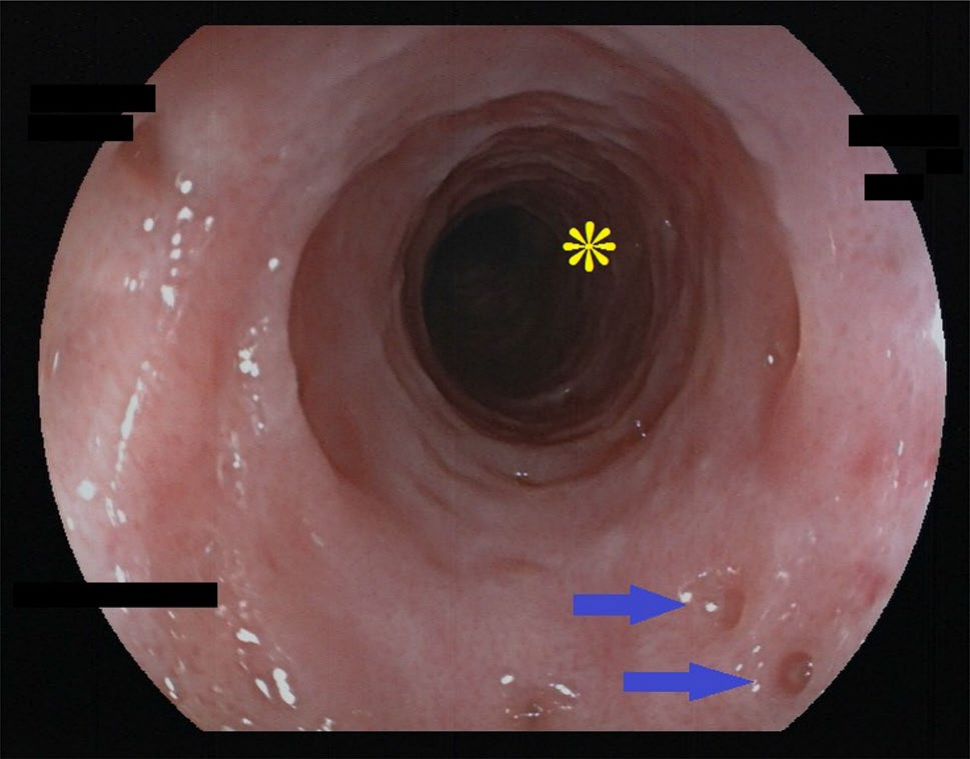
Trachealization of the esophagus. Overlap between faux uni pattern and frosted glass look. Pseudodiverticles in the foreground (arrows), multiple rings in the background (asterisk), rigid esophagus with narrow lumen and no peristalsis (“trachealization”). Patient 2 from Tables 1 and 2; Fujinon EG-600WR, VP-4450HD

Ring formation was frequent. 16 out of 21 patients (76%) had multiple, non-stenosing rings over the whole length of the esophagus, while 13 (62%) had a pronounced ring at or near the cardia. An esophagus with multiple rings was sometimes described as “rigid” by the endoscopist, with reduced peristalsis, and gave a “stiff” or “scarred” tactile feedback over the forceps when taking biopsies. The macroscopic aspect in these cases resembled the “trachealization” of the esophagus that can be seen in eosinophilic esophagitis (Fig. 5, Online Resource 3) (Table 2).

**Table 2:** Endoscopic findings in diffuse esophageal intramural pseudodiverticulosis

Patient No.	Number of EGD procedures	More than 4 diverticle openings	Openings aligned longitudinally	Frosted glass look	Faux uni pattern	Macroscopic candidosis	Multiple rings	Single ring
1	4	+	+	+	+	0	+	+
2	5	+	0	+	+	0	0	0
3	2	+	+	+	0	0	+	0
4	4	+	+	+	+	+	+	+
5	7	+	0	+	0	+	+	+
6	10	+	0	+	+	+	+	+
7	5	+	+	0	+	0	+	0
8	5	+	+	+	+	0	+	0
9	1	+	+	+	0	0	+	+
10	2	+	+	+	+	0	+	0
11	12	+	+	+	+	+	+	+
12	5	0	0	+	+	0	0	+
13	2	+	+	+	+	+	+	+
14	5	+	0	+	+	+	+	+
15	1	+	+	+	0	0	0	+
16	6	+	+	+	+	+	+	+
17	2	+	+	+	+	+	+	+
18	1	+	0	+	+	0	0	0
19	1	+	0	+	+	+	+	+
20	2	+	+	0	+	0	0	0
21	1	+	+	0	0	0	+	0
Total	83	20	14	18	16	9	16	13
%		95	67	86	76	43	76	62

**Fig. 6:**
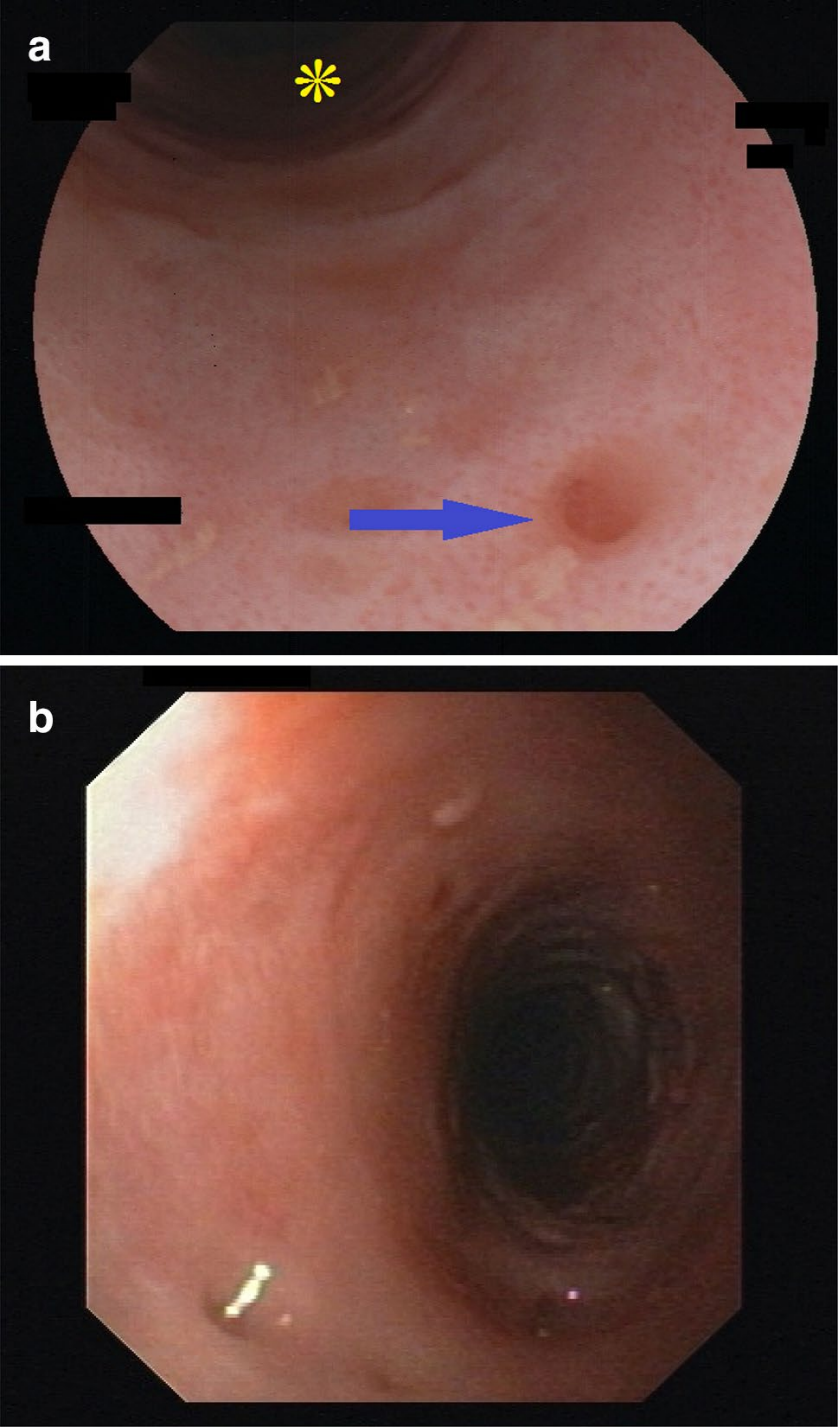
**a**: Closeup of faux uni pattern and small diverticle, high resolution. Endoscopic view of DEIPD, viewed with a high-definition zoom gastroscope/processor. Multiple small red dots, approximately 10–100 µm in diameter. Viewed from further away or with lower resolution, this will result in a pink appearance of the mucosa (“faux uni”). Small diverticle opening in the foreground, approximately 0.5 mm in diameter (arrow). Discrete ring formation and pink tint in the background (asterisk). Patient 2 from Tables 1 and 2; Fujinon EG-600WR, set to 1.5 × zoom, VP-4450HD. **b** Closeup of faux uni pattern and small diverticle, low resolution. Endoscopic view of DEIPD, viewed with an older, low-resolution gastroscope/processor. The small red dots clearly visible in **a** merge in to an evenly pink coloring of the mucosa; a pseudodiverticle opening in the foreground can easily be overlooked when the esophagus is passed too quickly. Patient 11 from Tables 1 and 2; Olympus GIF-160, Evis Exera II CLV-160/CV 160

Key endoscopic findings in DEIPD were multiple, longitudinally aligned diverticle openings, “frosted glass look” and “faux-uni” pattern of the mucosa. For definitions, see main text and Figs. 2, 3, 4, 5, 6

### Histology

**Fig. 7:**
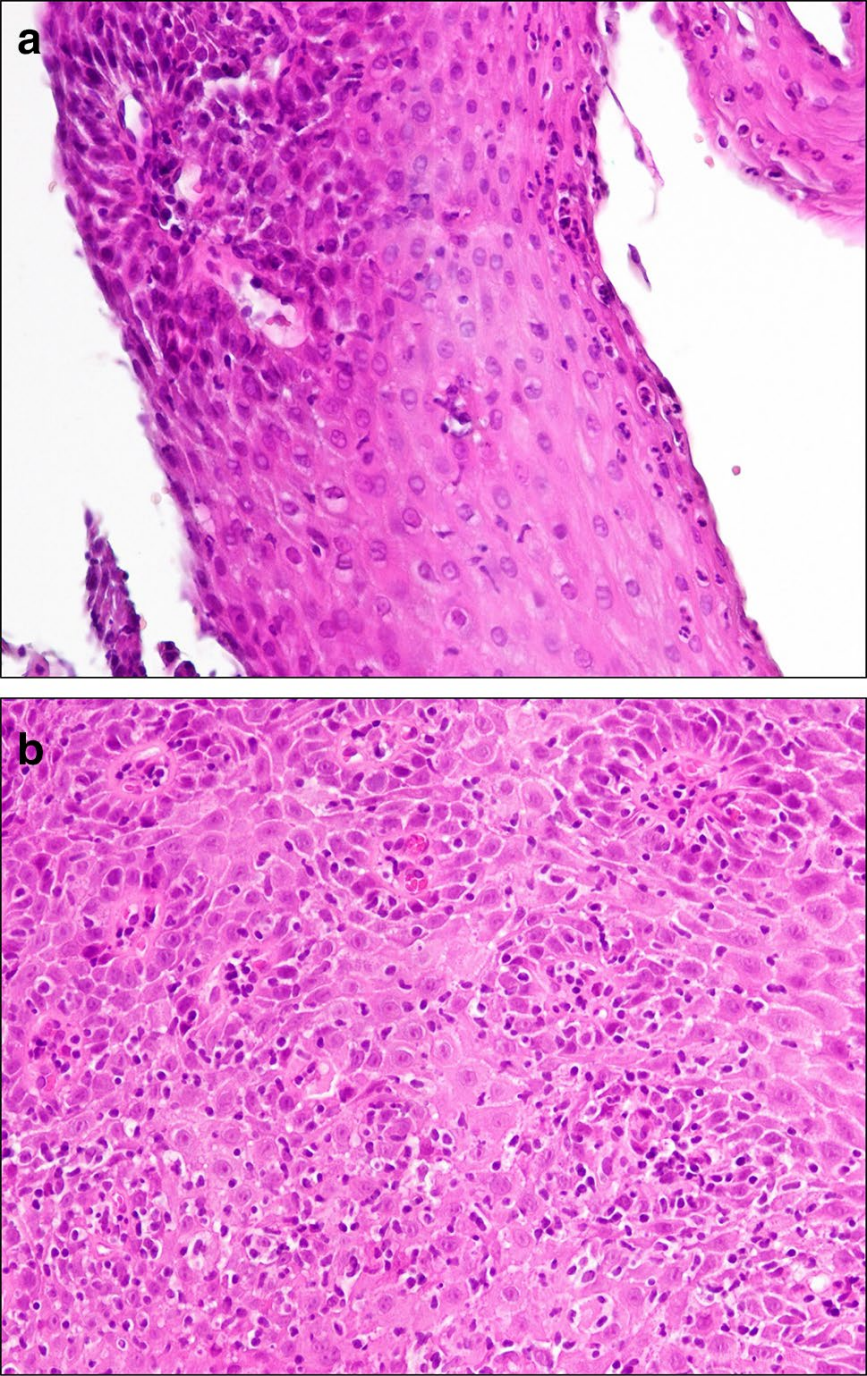
**a**: Histology in DEIPD. Esophageal biopsy in DEIPD. Mixed-cell type infiltrate, moderate chronic inflammatory fibrosis. Patient 12 from Tables 1 and 2, H&E staining, 200 ×. **b**: Histology in DEIPD. Esophageal biopsy in DEIPD. Mixed-cell type inflammation in and around the ducts of intramural glands. Patient 16 from Tables 1 and 2, H&E staining, 200 ×

In 19 out of 21 cases, mucosal biopsies were obtained. Sixteen of these showed unspecific inflammation, with a mixed infiltrate, inflammatory-reactive edema, squamous cell epithelial hyperplasia, and no signs of eosinophilic or lymphocytic esophagitis (Fig. 7a, b). Two cases showed moderate squamous cell hyperplasia and epidermization without acute inflammation.

In one case, eosinophils within the mixed infiltrate were reported as “increased” but not further quantified; eosinophilic abscesses were absent (Patient 19 from Tables 1 and 2). In another case, biopsy contained submucosa, but the stromal parts were too small to be of any diagnostic value (Patient 14). In jet another case, the pathologist suggested that epithelial hyperplasia may have led to ring formation and food transport interference (Patient 16).

## Discussion

### Epidemiology

DEIPD is a rare and poorly characterized disease. With only 250 published cases worldwide, its true incidence and prevalence are not known. In our own center that serves a population of roughly 150.000, we found 21 patients within 11 years. This is within the range recently reported by other centers of comparable size and hints to a prevalence in the order of magnitude between 5/100.000 and 50/100.000 [3, 4, 17]. It would mean that esophageal pseudodiverticulosis is still a rare disease but not as exceptional as previously estimated.

### Risk factors, symptoms

Alcohol and nicotine are suspected to be pathogenic factors [4, 14, 17]. In our own patients, the vast majority were males with a history of severe alcohol and/or tobacco abuse. Secondary diagnoses like liver cirrhosis, COPD, or atherosclerosis were present in 24%, 33%, and 38% of cases, respectively. Additionally, prevalence of non-esophageal malignancies and other relevant diagnoses like tuberculosis or pancreatitis was surprisingly high; mean hospitalization time was 2.7 days per patient and year (Fig. 1). We conclude that DEIPD preferably affects middle-aged men with pre-existing conditions and a pronounced risk profile, although its exact pathomechanism is unclear.

Many authors report severe, chronic dysphagia to be key symptom of DEIPD [17, 21, 23]. Our own data partly support that. About two-thirds of our patients presented with dysphagia as initial symptom or developed it later on, but one-third never had dysphagia at all. On the other hand, about half of our patients presented with food impaction at least once. One patient had five episodes of food impaction within 32 months (Table 1). This is substantially more than in any other series and reviews [3, 4, 17, 21]. However, a look at case reports published so far reveals that food impaction may be more common in DEIPD than previously thought [24–26]. Since chronic dysphagia often leads to weight loss, and since food impaction usually presents as an endoscopic emergency, it shows the significance of this disease.

### Endoscopy

Before the introduction of high-resolution video endoscopy, DEIPD was diagnosed according to well-defined radiologic criteria [2, 16, 27]. Since then, however, there has been no detailed description of endoscopic-macroscopic signs of the disease. Our own findings suggest the following endoscopic criteria to be pathognomonic for DEIPD:

From histopathologic studies it is known that esophageal pseudodiverticles are formed by dilated excretory ducts of submucosal glands. We, therefore, propose an opening size of 0.25–5 mm as a key criteria for DEIPD, because it reflects the size of an enlarged and inflamed duct of these glands [28]. We furthermore propose a cutoff value of five or more diverticle openings in the esophageal wall because this number was reached in 95% of patients. A longitudinal alignment of diverticles was seen in two-thirds of cases and reflects the longitudinal alignment of normal mucosal glands known from micro-anatomic studies [29] (Fig. 2, Online Resource 1) (Table 2).

Another macroscopic characteristic of DEIPD is the diffuse, dull-white, supposedly edematous, swelling of the mucosa around diverticle openings. It is often accompanied by active inflammation and oozing of liquid. In the absence of a better phrasing, we propose the term “frosted glass look” for this phenomenon (Figs. 4 and 5, Online Resource 5).

One more sign of DEIPD previously undescribed is the phenomenon we termed as “faux uni pattern”. Until the turn of the century, fiber-optic endoscopes had a typical optical resolution of 2–4 lp/mm. First electronic scopes reached 5–8 lp/mm and were connected to vacuum-tube tube video screens, or 800 × 600 pixel LEDs [30]. Detecting mucosal dots smaller than 125–250 µm in diameter was virtually impossible with that technology. Therefore, mucosa in DEIPD appeared uniquely pink. Only with today’s high-definition endoscopes [31] can the pattern of tiny red dots that form this pink tint be observed directly (Figs. 3, 6a, b, Online Resource 1, 2, and 4). Albeit present in three quarters of our patients, faux-uni dot pattern is not exclusive for DEPID. After years of chronic reflux esophagitis, it can sometimes be seen in the lower esophagus as well. We, therefore, suspect it to be a residual state after longstanding inflammation. Frosted glass look, on the other hand, seems to be a sign of active inflammation.

The majority of our patients had multiple esophageal rings (Table 2). Like faux uni pattern, these are typical but not exclusive for DEIPD. Rings can also be seen as a result of other diseases like EoE or lymphocytic esophagitis (LyE) [32, 33]. They are thought to be a form of submucosal scarring and fibrosis due to chronic inflammation—a phenomenon called “trachealization” of the esophagus (Fig. 5, Online Resource 3) [34, 35].

Functionally, inflammation and destruction of submucosal glands will lead to impaired lubrication, sclerosis, and trachealization to impaired peristalsis [32, 36, 37]. Both phenomena together explain the high rate of dysphagia and food impactions in DEIPD patients.

### Histology

Histopathologic studies on diffuse esophageal pseudodiverticulosis are scarce; what we know mostly derives from autopsy findings or esophagectomy specimens [1, 2, 38]. At mucosal level, a mixed-cell type inflammation is typical. Albeit unspecific, this discriminates the disease from the highly pathognomonic changes in eosinophilic esophagitis or lymphocytic esophagitis [39, 40]. In our own patients, we could reproduce these findings in 16 out of 19 mucosal biopsies (84%) showing a mixed leuko-lymphocytic mucosal infiltrate and no increased eosinophils (Fig. 7a, b).

Additionally, many of our patients had reactive squamous cell hyperplasia and/or edema. It can be hypothesized that this might possibly reflect the “faux uni pattern” and “frosted glass look” we saw macroscopically.

Fibrosis in DEIPD occurs around the esophageal glands, which are located mainly in the submucosa [1, 2]. In our own patients, we found fibrosing inflammation only in a fraction of mucosal biopsies but that does not rule out fibrosis of deeper layers. Unfortunately, systematic assessment of these layers would have required deep transmucosal biopsies, which are hazardous and ethically questionable and, therefore, not routinely obtained [41]. We can on only speculate that circular fibrosis may contribute to ring formation and that these rings form at the site of maximum chronic inflammation. In GERD, this will typically be the lower esophagus sphincter, and in DEIPD (as in EoE) it can be anywhere.

## Conclusion

One aim of our study was to establish a pathway to the diagnosis of DEIPD. Risk factors like male sex, alcohol- and tobacco abuse were unspecific. Clinical symptoms like dysphagia and bolus impaction were typical, but also too unspecific to be pathognomonic (Table 1). In endoscopy, however, we found a combination of macroscopic criteria that we think is highly indicative for DEIPD (Table 2, List Box 1) (Online Resource 6). Once the suspicion is raised, we recommend mucosal biopsies, mainly to rule out the two most likely differential diagnoses—EoE and lymphocytic esophagitis.

**List Box 1:** Endoscopic findings in diffuse esophageal intramural pseudodiverticulosis

• 5 or more small (0.25–5 mm) pseudodiverticle openings
• Openings aligned longitudinally
• Edematous swelling of mucosa—“frosted glass look”
• Fine-grained spotty reddening of mucosa—“faux uni pattern”
• Multiple rings, rigid esophagus (“trachealization”)
• Distal third not more affected than rest of esophagus
• No other cause for diverticles (Zenker’s, pulsion diverticle, connective tissue disorders, …)

## Limitations

Our study has flaws. First, since it roots in a retrospective database search, there is a principal risk of selection bias. We have tried to counteract this by relating the 21 cases not to the number of patients in our database but to the overall population. Second, as in many works on rare diseases, the small number of cases prohibits the use of inferential statistics. We, therefore, present our raw data (Tables 1 and 2) and applied only descriptive statistics. As a result, it is not possible to assess the significance of our findings. Especially when applying our endoscopic criteria in a clinical context, one has to be aware that their sensitivity and specificity are not formally defined. Finally, we have consciously limited this study to clinical and endoscopic phenomena. Histopathology was mainly used to rule out differential diagnoses, pathogenetic and therapeutic aspects were omitted. Further work in these fields is required and will hopefully lead to a better understanding of diffuse esophageal intramural pseudodiverticulosis.

## Electronic supplementary material

Electronic supplementary material 1 (TXT 1 kb)

Electronic supplementary material 2 (JPG 106 kb)

Electronic supplementary material 3 (JPG 106 kb)

Electronic supplementary material 4 (JPG 114 kb)

Electronic supplementary material 5 (JPG 102 kb)

Electronic supplementary material 6 (JPG 98 kb)

Electronic supplementary material 7 (JPG 94 kb)

## Abbreviations

DEIPD: Diffuse esophageal intramural pseudo-diverticulosis
EGD: Esophago-gastro-duodenoscopy
EIPD: Esophageal intramural pseudo-diverticulosis (used sysnonymusly)
EoE: Eosinophilic esophagitis
GERD: Gastroesophageal reflux disease
H&E: Hematoxilin & eosin
lp/mm: Line points per millimeter
SD: Standard deviation

## References

[1] Medeiros LJ, Doos WG, Balogh K (1988). Esophageal intramural pseudodiverticulosis: a report of two cases with analysis of similar, less extensive changes in "normal" autopsy esophagi. Hum Pathol.

[2] Wightman AJ, Wright EA (1974). Intramural oesophageal diverticulosis: a correlation of radiological and pathological findings. Br J Radiol.

[3] Bechtler M, Vollmer H, Vetter S (2014). Long-term follow-up after dilation in symptomatic esophageal intramural pseudodiverticulosis: an observational study in 22 cases. Endoscopy.

[4] Halm U, Lamberts R, Knigge I (2014). Esophageal intramural pseudodiverticulosis: endoscopic diagnosis and therapy. Dis Esophagus.

[5] Montgomery RD, Mendl K, Stephenson SF (1975). Intramural diverticulosis of the oesophagus. Thorax.

[6] Yamamoto N, Nakamura M, Tachibana S (2002). Esophageal intramural pseudodiverticulosis with Mallory-Weiss syndrome: report of a case. Surg Today.

[7] Chino O, Makuuchi H, Kondo Y (2014). Esophageal intramural pseudodiverticulosis treated by endoscopic balloon dilatation. Tokai J Exp Clin Med.

[8] Tyberg A, Jodorkovsky D (2014). A treatment option for esophageal intramural pseudodiverticulosis. ACG Case Rep J.

[9] Chiba T, Iijima K, Koike T (2012). A case of severe esophageal intramural pseudodiverticulosis whose symptoms were ameliorated by oral administration of anti-fungal medicine. Case Rep Gastroenterol.

[10] Akkari I, Ben Jazia E, Mrabet S (2019). Candida albicans: a cause or a consequence of esophageal intramural pseudo-diverticulosis. Pan Afr Med J.

[11] Mendl K, McKay JM, Tanner CH (1960). Intramural diverticulosis of the oesophagus and Rokitansky-Aschoff sinuses of the gall-bladder. Br J Radiol.

[12] Fee BE, Dvorak AD (1976). Intramural pseudodiverticulosis of the esophagus. Neb Med J.

[13] Brühlmann WF, Zollikofer CL, Maranta E (1981). Intramural pseudodiverticulosis of the esophagus: report of seven cases and literature review. Gastrointest Radiol.

[14] Sabanathan S, Salama FD, Morgan WE (1985). Oesophageal intramural pseudodiverticulosis. Thorax.

[15] Plavsic BM, Chen MY, Gelfand DW (1995). Intramural pseudodiverticulosis of the esophagus detected on barium esophagograms: increased prevalence in patients with esophageal carcinoma. AJR Am J Roentgenol.

[16] Levine MS, Moolten DN, Herlinger H (1986). Esophageal intramural pseudodiverticulosis: a reevaluation. AJR Am J Roentgenol.

[17] Scaffidi MA, Garg A, Ro B (2016). Esophageal intramural pseudodiverticulosis and concomitant eosinophilic esophagitis: a case series. Can J Gastroenterol Hepatol.

[18] Takeshita N, Kanda N, Fukunaga T (2015). Esophageal intramural pseudodiverticulosis of the residual esophagus after esophagectomy for esophageal cancer. World J Gastroenterol.

[19] Naoi Y, Katayama H, Tomiyoshi H (1997). Esophageal intramural pseudodiverticulosis with esophageal cancer improved by target rotation irradiation: case report. Nihon Igaku Hoshasen Gakkai Zasshi.

[20] Farack UM, Kinnear DG, Jabbari M (1979). Intramural pseudodiverticulosis of the esophagus—a primarily radiologic diagnosis. Rofo.

[21] Hahne M, Schilling D, Arnold JC (2001). Esophageal intramural pseudodiverticulosis: review of symptoms including upper gastrointestinal bleeding. J Clin Gastroenterol.

[22] Riphaus A, Wehrmann T, Hausmann J (2015). S3-guidelines “sedation in gastrointestinal endoscopy” 2014 (AWMF register no. 021/014). Z Gastroenterol.

[23] Koyama S, Watanabe M, Iijima T (2002). Esophageal intramural pseudodiverticulosis (diffuse type). J Gastroenterol.

[24] Schmutz G, Zeller C, Doffoel M (1983). Une cause rare de blocage alimentaire. La pseudo-diverticulose intra-murale de l'oesophage. Presse Med.

[25] Eliakim R, Libson E, Rachmilewitz D (1989). Diffuse intramural esophageal pseudodiverticulosis. J Natl Med Assoc.

[26] Attila T, Marcon NE (2006). Esophageal intramural pseudodiverticulosis with food impaction. Can J Gastroenterol.

[27] Canon CL, Levine MS, Cherukuri R (2000). Intramural tracking: a feature of esophageal intramural pseudodiverticulosis. AJR Am J Roentgenol.

[28] Yagi K, Nakamura A, Sekine A, Umezu H (2006). The prevalence of esophageal cardiac glands: relationship with erosive esophagitis and nonerosive reflux disease (NERD) in Japanese patients. Endoscopy.

[29] Zhang X, Patil D, Odze RD (2018). The microscopic anatomy of the esophagus including the individual layers, specialized tissues, and unique components and their responses to injury. Ann N Y Acad Sci.

[30] Seidlitz HK, Classen M (1992). Optical resolution and color performance of electronic endoscopes. Endoscopy.

[31] Sivak MV (2006). Gastrointestinal endoscopy: past and future. Gut.

[32] Chen JW, Pandolfino JE, Lin Z (2016). Severity of endoscopically identified esophageal rings correlates with reduced esophageal distensibility in eosinophilic esophagitis. Endoscopy.

[33] Pleet JL, Taboada S, Rishi A (2017). Rings in the esophagus are not always eosinophilic esophagitis: case series of ring forming lymphocytic esophagitis and review of the literature. Endosc Int Open.

[34] Al-Hussaini AA, Semaan T, El Hag IA (2009). Esophageal trachealization: a feature of eosinophilic esophagitis. Saudi J Gastroenterol.

[35] Nandy N, Rustagi T (2019). “Trachealization” of the esophagus. N Engl J Med.

[36] Sarosiek J (2016). Does the healing of the esophageal mucosa improve the function of the esophageal submucosal and salivary glands?. Ann N Y Acad Sci.

[37] Sarosiek J, McCallum RW (1995). What is the secretory potential of submucosal mucous glands within the human gullet in health and disease?. Digestion.

[38] Tsuboi J, Tajika M, Nakamura T (2010). Endoscopic features of short-term progression of esophageal intramural pseudodiverticulosis. Endoscopy.

[39] Dellon ES (2012). Eosinophilic esophagitis: diagnostic tests and criteria. Curr Opin Gastroenterol.

[40] Patil DT, Hammer S, Langer R, Yantiss RK (2018). Lymphocytic esophagitis: an update on histologic diagnosis, endoscopic findings, and natural history. Ann N Y Acad Sci.

[41] Armbruster-Lee J, Cavender CP, Lieberman JA, Samarasinghe AE (2018). Understanding fibrosis in eosinophilic esophagitis: are we there yet?. J Leukoc Biol.

