# Supplementary figures and images for: Clinical and endoscopic characteristics of diffuse esophageal intramural pseudo-diverticulosis
Source: Esophagus. 2020 Mar 11;17(4):492–501. doi: 10.1007/s10388-020-00729-6 (PMC7497296; doi:10.1007/s10388-020-00729-6)

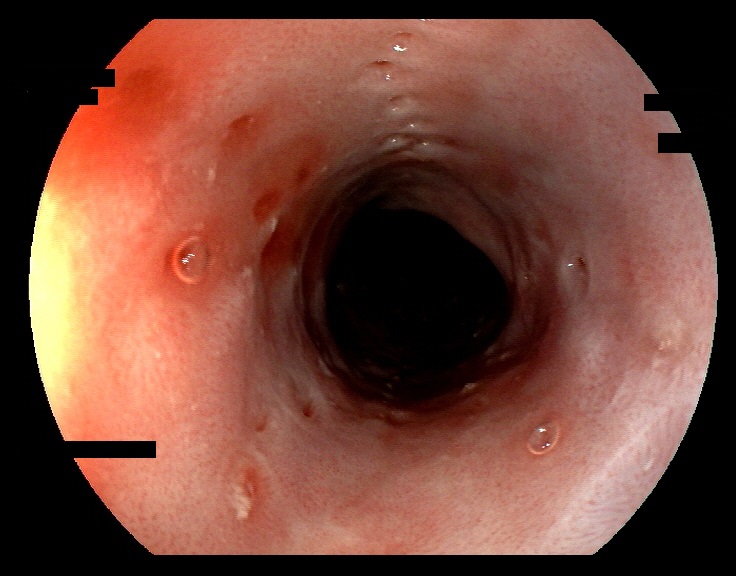

Supplement: Supplementary file 2 — Electronic supplementary material 2 (JPG 106 kb) [file 10388_2020_729_MOESM2_ESM.jpg]

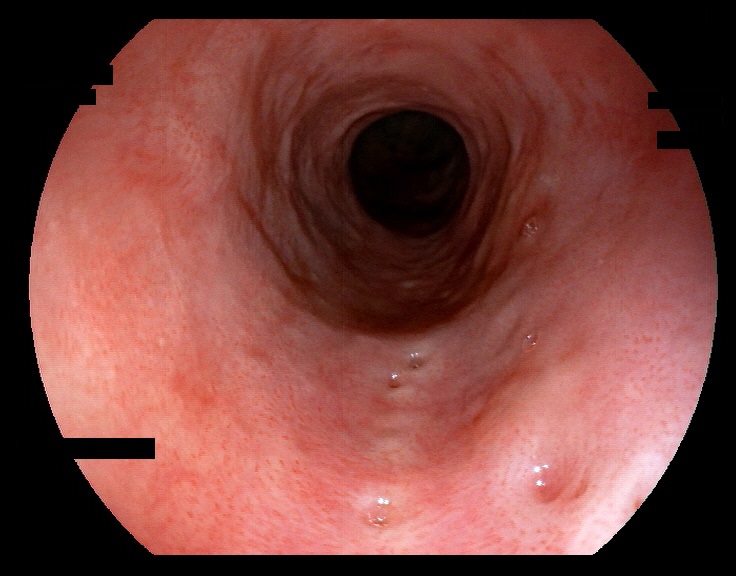

Supplement: Supplementary file 3 — Electronic supplementary material 3 (JPG 106 kb) [file 10388_2020_729_MOESM3_ESM.jpg]

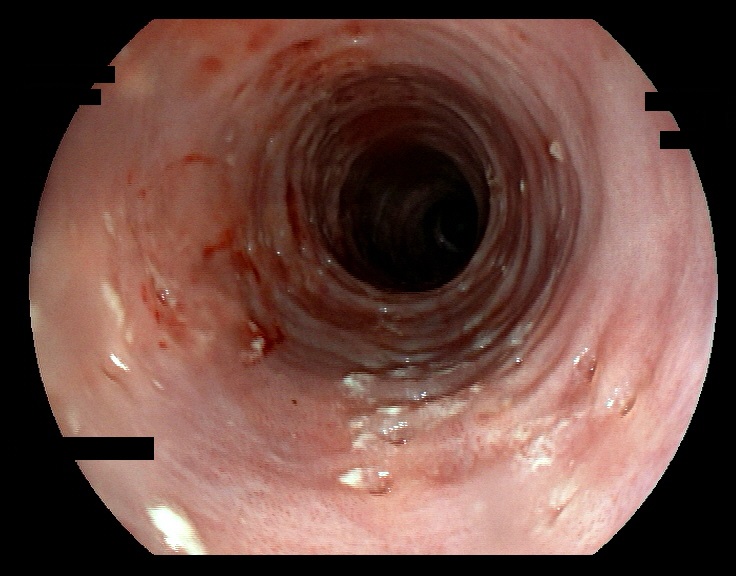

Supplement: Supplementary file 4 — Electronic supplementary material 4 (JPG 114 kb) [file 10388_2020_729_MOESM4_ESM.jpg]

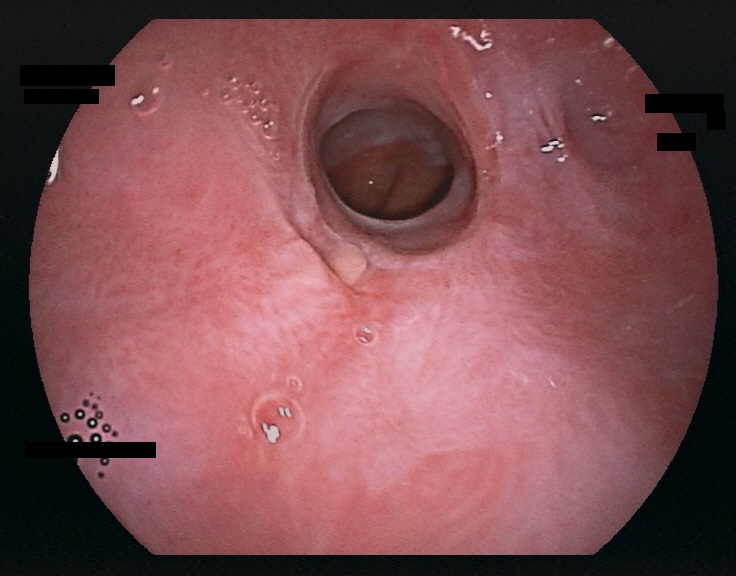

Supplement: Supplementary file 5 — Electronic supplementary material 5 (JPG 102 kb) [file 10388_2020_729_MOESM5_ESM.jpg]

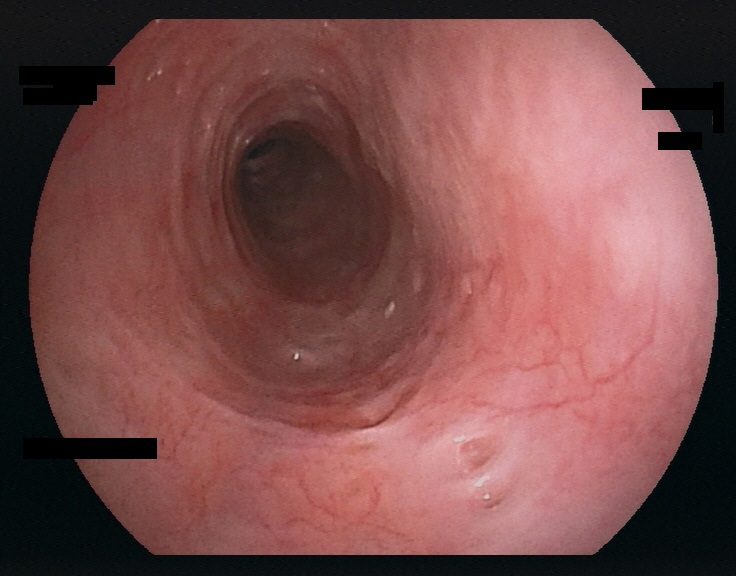

Supplement: Supplementary file 6 — Electronic supplementary material 6 (JPG 98 kb) [file 10388_2020_729_MOESM6_ESM.jpg]

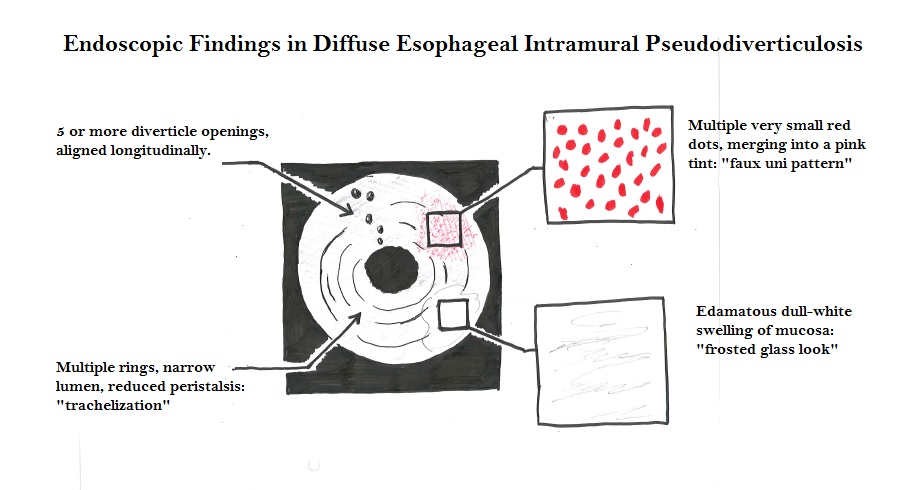

Supplement: Supplementary file 7 — Electronic supplementary material 7 (JPG 94 kb) [file 10388_2020_729_MOESM7_ESM.jpg]
